# Optimal strategies for umbilical cord mesenchymal stem cell–derived exosomes in acute kidney injury: a network meta-analysis in rat models

**DOI:** 10.3389/fcell.2026.1794218

**Published:** 2026-04-28

**Authors:** Pingping Wanyan, Nenglian Li, Linna Ma, Li Zhang

**Affiliations:** Department of Pathology and Pathophysiology, Gansu University of Chinese Medicine, Lanzhou, Gansu, China

**Keywords:** acute kidney injury, dosing and delivery strategy, exosomes, network meta-analysis, umbilical cord mesenchymal stem cells

## Abstract

**Background:**

Exosomes derived from umbilical cord mesenchymal stem cells (UCMSC-Exos) have emerged as a highly promising cell-free therapeutic strategy for repairing acute kidney injury (AKI). However, preclinical evidence regarding their efficacy and optimal administration strategy remains heterogeneous and has not been systematically synthesized.

**Methods:**

PubMed, Web of Science, Embase, and Scopus were systematically searched to identify randomized controlled experiments evaluating UCMSC-Exos in rat models of AKI. Conventional meta-analyses were performed to pool effect sizes, and frequentist network meta-analyses were used to compare the relative efficacy and ranking probabilities of different interventions.

**Results:**

Fourteen studies were included. Conventional meta-analysis showed that, compared with controls, UCMSC-Exos significantly reduced serum creatinine (Scr; SMD = −5.60, 95% CI: −7.61 to −3.60) and blood urea nitrogen (BUN; SMD = −6.33, 95% CI: −8.91 to −3.75), alleviated renal histological injury scores (SMD = −3.62, 95% CI: −4.91 to −2.), and decreased cell apoptosis (SMD = −4.35, 95% CI: −6.06 to −2.64). Network meta-analysis further indicated that, at a fixed dose of 100 μg, tail vein injection was significantly superior to subcapsular renal injection in reducing Scr (SUCRA: 94.2% vs. 55.8%) and showed a similar trend for BUN. Within each administration route, distinct dose–response patterns were observed. For tail vein injection, there were no significant differences in efficacy among 30 μg, 100 μg, and 250 μg, although 100 μg showed the most favorable trend. In contrast, subcapsular renal injection exhibited a dose-dependent pattern, with higher doses (200 μg, 400 μg) being more effective than the lower 100 μg dose. Methodological reporting quality was generally inadequate, and potential publication bias was detected; however, the adjusted effect size remained statistically significant (SMD = −2.59).

**Conclusion:**

UCMSC-Exos effectively improve renal function, histopathology, and cell survival in rat models of AKI. Tail vein injection, particularly at a dose of 100 μg, may represent the most effective strategy, whereas subcapsular renal injection may require higher doses to achieve adequate efficacy. These conclusions should be interpreted cautiously, and future studies should more rigorously control for and report potential confounders, such as animal sex and AKI induction methods.

## 1 Introduction

Acute kidney injury (AKI) is a common yet serious clinical syndrome characterized by an abrupt decline in glomerular filtration rate, leading to azotemia, electrolyte imbalances, and fluid and sodium retention (Ostermann et al., 2025). AKI not only prolongs hospitalization and increases costs, but also markedly elevates both short- and long-term mortality. Epidemiological data indicate that more than 13 million individuals develop AKI annually worldwide, approximately 85% of whom are in low- and middle-income countries; the condition affects roughly one-fifth of hospitalized adults and 50%–60% of patients in intensive care units (Cerda et al., 2025). Despite advances in renal replacement therapy and critical care, overall mortality from AKI has not declined substantially over the past 2 decades (Ostermann et al., 2025). Given its heterogeneous etiologies and multifaceted pathogenesis, no specific pharmacotherapy has yet been proven to improve prognosis, and current clinical management remains largely supportive (Ostermann et al., 2025; Ostermann et al., 2024).

Stem cell–based therapy, an advanced regenerative strategy, has attracted considerable attention for renal repair in recent years. In particular, umbilical cord–derived mesenchymal stem cells (UC-MSCs) and their derivatives have shown distinct advantages in tissue regeneration and immunomodulation. UC-MSCs are readily available, free of major ethical concerns, exhibit low immunogenicity, and can modulate repair in injured tissues through paracrine mechanisms (Mebarki et al., 2021; Han et al., 2025). Among these secreted products, exosomes—nanometer-scale extracellular vesicles carrying proteins and nucleic acids—mediate intercellular communication and exert diverse biological effects, including immune regulation, anti-apoptosis, and pro-angiogenesis (Liu and Wang, 2023; Kaur et al., 2024). These features endow UC-MSC–derived exosomes (UCMSC-Exo) with substantial therapeutic potential for the treatment of AKI. A growing body of preclinical research using rat models has evaluated the efficacy and mechanisms of UCMSC-Exo in AKI (Oz Oyar et al., 2022; Wang et al., 2017; Wan et al., 2023). However, marked heterogeneity exists across studies with respect to experimental design, injury models, administration routes, dosing, timing, and outcome assessments, limiting comparability and hindering optimization and translation of exosome-based therapies. High-quality evidence synthesizing these data remains lacking.

Accordingly, based on rat models of AKI, this study, for the first time, combines conventional meta-analysis with network meta-analysis to quantitatively synthesize the therapeutic effects of UC-MSC–derived exosomes on renal structural and functional recovery. Our objectives are to assess the overall impact of UCMSC-Exo on key AKI endpoints and to compare the relative efficacy of different dosing regimens and delivery routes, and thereby to identify optimal application strategies in experimental animals. We aim to provide a rigorous evidence base for preclinical development of exosome therapy for AKI and to inform its future clinical translation and individualized application.

## 2 Methods

### 2.1 Study protocol and registration

This review was prospectively registered with the International Prospective Register of Systematic Reviews (PROSPERO). The conduct and reporting adhered to the Preferred Reporting Items for Systematic Reviews and Meta-Analyses (PRISMA) guidelines and followed SYRCLE guidance for the methodological appraisal of animal studies.

### 2.2 Eligibility criteria

#### 2.2.1 Study design

We included randomized controlled experiments (RCTs) conducted in rat models, irrespective of whether allocation concealment or blinding was implemented.

#### 2.2.2 Study subjects

We included randomized controlled experiments (RCTs) conducted in rat models, irrespective of whether allocation concealment or blinding was implemented.

#### 2.2.3 Study subjects

Rats were chosen as the target species because they are the most widely used animal model in AKI research. Our preliminary scoping search indicated that studies in mice are limited in number and highly heterogeneous. More importantly, rats and mice differ substantially in genetic background, immune–metabolic profiles, and mechanisms of renal injury and repair; forcing a pooled analysis of the two species would introduce uncontrollable heterogeneity and violate the homogeneity assumption of meta-analysis. For this reason, the present review restricted inclusion to rat studies to avoid confounding from interspecies differences. No restrictions were placed on rat strain, sex, or age. Included AKI models were required to have clearly specified induction methods. Studies involving non-rat species or models with concomitant chronic kidney disease, renal tumors, or autoimmune kidney diseases were excluded to avoid confounding from species differences and pre-existing renal pathology on the assessment of UCMSC-Exo efficacy.

#### 2.2.4 Interventions and comparators

The intervention consisted of purified umbilical cord mesenchymal stem cell–derived exosomes (UCMSC-Exo). Studies were required to report the single dose or cumulative treatment dose and the delivery route (e.g., tail-vein injection, targeted renal arterial injection, intraperitoneal injection). Comparators included: (i) blank controls (AKI induced with no subsequent intervention), (ii) vehicle controls (the solvent system used for UCMSC-Exo, such as sterile PBS or serum-free DMEM), and (iii) active controls differing by dose and/or route (e.g., low-vs. high-dose UCMSC-Exo, intravenous vs. renal arterial administration).

#### 2.2.5 Outcomes

Outcome measures were required to provide extractable quantitative data, including serum creatinine (Scr) and blood urea nitrogen (BUN), measured using fully automated biochemical analyzers by enzymatic or colorimetric methods, with values directly reflecting the severity of renal functional impairment; renal tissue injury scores, assessed using internationally recognized AKI histopathological scoring systems (e.g., tubular injury score, Paller score) based on H&E− or PAS-stained sections to evaluate the extent of tubular epithelial necrosis, tubular dilatation and cast formation, and the degree of interstitial inflammatory cell infiltration, with higher scores indicating more severe injury; and TUNEL-positive cell counts, determined by fluorescence-labeled TUNEL staining and expressed as the number of positive cells per high-power field (HPF) or the percentage of positive cells, with values reflecting the level of apoptosis in renal tissue.

#### 2.2.6 Exclusion criteria

We excluded studies with unavailable full texts or missing outcome data; unclear UCMSC-Exo dose or route; exosomes not derived from UC-MSCs; crossover or contralateral designs that confound allocation (e.g., assigning the two kidneys of the same rat to different arms, or pre-/post-self controls); non-RCT designs (e.g., self-controlled before–after studies); secondary research (reviews, meta-analyses, or commentaries); gray literature (conference abstracts, preprints, or case reports); and purely *in vitro* experiments.

### 2.3 Search strategy

We searched PubMed, Web of Science, Embase, and Scopus from inception to 7 October 2025. Search strategies combined controlled vocabulary and free-text terms, tailored to each database. Core English terms included “umbilical cord mesenchymal stromal cell exosomes,” “exosomes,” “extracellular vesicles,” and “acute kidney injury.” We also screened reference lists of included studies to minimize omissions. Full search strategies are provided in Supplementary Table S1.

### 2.4 Study selection and data extraction

Two researchers trained in systematic review methodology independently screened the literature and extracted data, with disagreements resolved through discussion or adjudication by a third methodologist specializing in statistical methods. The screening process consisted of an initial screening (title and abstract) to exclude obviously ineligible records, followed by a full-text review to strictly assess compliance with the inclusion criteria. The screening process and reasons for exclusion are presented using the PRISMA 2020 flow diagram. During the screening process, we first used EndNote software and manual checks to remove duplicate records. For studies published around the same time from the same research group or institution, we performed a detailed comparison of key parameters—such as author information, animal characteristics (strain, body weight, model induction method), interventions (exosome source, dose, route), and sample size—to identify any data splitting or overlapping publications based on the same experimental animal cohort. Data extraction was performed using a standardized Excel form that was pre-designed and pilot-tested. The extracted information included: basic study characteristics (first author, year, country); animal baseline and model information (strain, body weight, model induction method); UCMSC-Exo details (dose, route, timing of administration); prespecified outcome measures; and methodological quality-related information (random sequence generation, allocation concealment, and blinding procedures).

### 2.5 Risk-of-bias assessment

Methodological quality was appraised using the SYRCLE risk-of-bias tool, adapted for AKI animal experiments. Domains included: random sequence generation (e.g., random number tables, computer-generated sequences); allocation concealment (e.g., sealed opaque envelopes, centralized allocation); baseline comparability (balance of group characteristics); random housing; blinding of caregivers/investigators; blinded outcome assessment (pathology scoring and laboratory assays); incomplete outcome data (reporting and handling of attrition/missingness); selective reporting (consistency with protocol and completeness of prespecified outcomes); and other biases (e.g., sample-size calculation, conflicts of interest). Each domain was rated as “low risk (yes),” “high risk (no),” or “unclear risk (insufficient information),” and results were visualized as a risk-of-bias heatmap.

### 2.6 Statistical analysis

Statistical analyses were performed using R software (version 4.4.2) and STATA (version 16.0). All tests were two-sided with a significance level of α = 0.05, and differences were considered statistically significant when the 95% confidence interval (95% CI) of the effect size did not include 0. All outcomes were continuous variables, and standardized mean differences (SMD) were used to pool effect sizes to eliminate the impact of differences in assay methods and measurement units and, thereby, to partially mitigate the influence of varying injury severity across models.

In the head-to-head meta-analysis, heterogeneity was assessed using Cochran’s Q test and the I^2^ statistic. When I^2^ ≤ 50% and the Q test yielded P ≥ 0.10, a fixed-effect model was applied for pooling. When I^2^ > 50% or the Q test gave P < 0.10, a random-effects model (with between-study variance τ^2^ estimated using restricted maximum likelihood) was used, and sources of heterogeneity were explored through subgroup analyses stratified by UCMSC-Exo dose and administration route. Publication bias was assessed for outcomes with 10 or more included studies using visual inspection of funnel plot symmetry, combined with Egger’s linear regression (to quantitatively test for publication bias) and Begg’s rank correlation test. If bias was identified, the trim-and-fill method was applied to adjust and re-pool the effect sizes.

The frequentist network meta-analysis was conducted within a generalized linear mixed-model framework. We first fit a consistency model to obtain combined effects, then examined agreement between direct and indirect evidence using node-splitting and loop-specific inconsistency tests; P < 0.05 indicated significant inconsistency, in which case an inconsistency model was applied. We estimated direct, indirect, and network (combined) effects for each comparison, reporting SMDs with 95% CIs to contrast the relative efficacy of different doses and routes. Treatments were ranked using the surface under the cumulative ranking curve (SUCRA) and cumulative ranking plots, with higher SUCRA values indicating greater relative efficacy. Heterogeneity was summarized by τ^2^. Robustness was assessed by comparing results from consistency and inconsistency models to ensure the reliability of the network meta-analytic conclusions.

## 3 Results

### 3.1 Literature search

**FIGURE 1:**
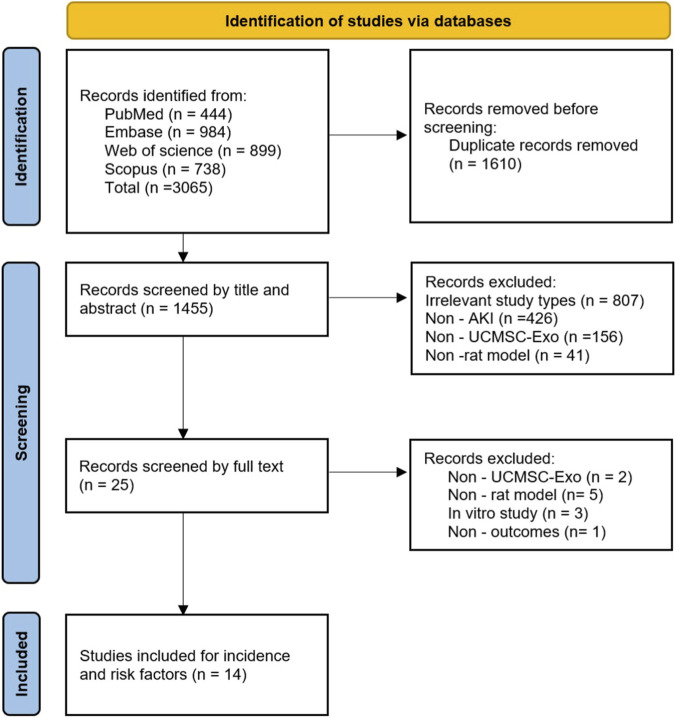
PRISMA flow diagram.

A total of 3,065 relevant records were identified in the initial search. After removing 1,610 duplicates, the titles and abstracts of 1,455 records were screened. Following this screening, 1,430 records that did not meet the eligibility criteria were excluded. The remaining 25 records underwent full-text assessment, and 11 studies were further excluded for the following reasons: non-UCMSC-Exo interventions (n = 2), non-rat models (n = 5), *in vitro* studies (n = 3), and absence of relevant outcomes (n = 1). Ultimately, 14 studies were included in the network meta-analysis (Oz Oyar et al., 2022; Wang et al., 2017; Wan et al., 2023; Zhou et al., 2013; Zhang et al., 2014; Zou et al., 2014; Ju et al., 2015; Gu et al., 2016; Zhang et al., 2016; Zou et al., 2016a; Zou et al., 2016b; Chen et al., 2017; Jia et al., 2018; Sadek et al., 2023). The study selection process is depicted in Figure 1.

### 3.2 Characteristics of included studies

All 14 included studies were randomized controlled animal experiments (RCTs), published between 2013 and 2023. After verifying author teams, animal characteristics, and experimental designs, all studies were confirmed to be based on independent experimental animal cohorts, with no data duplication or overlapping publications. Rat strains comprised Sprague–Dawley rats (12 studies), Wistar rats (1 study), and Albino rats (1 study). Ten studies used male animals and four used female animals. Body weight ranged from 180 to 250 g. Six studies reported rat ages between 6 and 8 weeks, while the remaining eight did not report age. Sample sizes per group ranged from 6 to 24, with a total of 176 experimental animals included. AKI were mainly induced by cisplatin (4 studies) or by ischemia–reperfusion injury (10 studies). Within the ischemia–reperfusion injury models, specific procedures were heterogeneous: 4 studies used unilateral ischemia–reperfusion, 2 used bilateral ischemia–reperfusion, and 4 used unilateral ischemia–reperfusion combined with contralateral nephrectomy. All studies used UCMSC-Exos as the intervention. The primary routes of administration were tail vein injection (9 studies) and subcapsular renal injection (5 studies). Doses ranged from 30 to 400 μg, with 100 μg being the most commonly used dose (9 studies). Control groups mainly received normal saline, PBS, or various culture media. The basic characteristics of the included studies are summarized in Supplementary Table S2.

### 3.3 Risk of bias

**FIGURE 2:**
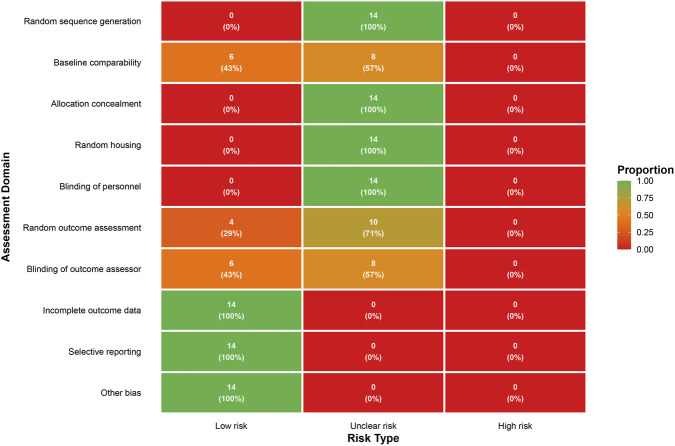
Risk-of-bias assessment for the included studies.

For “sequence generation,” “allocation concealment,” “random housing,” and “blinding of caregivers/investigators,” all 14 studies were judged as “unclear risk.” Baseline comparability was rated “low risk” in 6 studies, while 8 were “unclear” due to insufficient reporting. Regarding outcome assessment, “random outcome assessment” (i.e., random selection of animals for outcome evaluation) was “low risk” in 4 studies and “unclear” in 10; blinding of outcome assessors was “low risk” in 6 and “unclear” in 8. With respect to reporting, all studies were judged “low risk” for incomplete outcome data, selective reporting, and other biases (Figure 2).

### 3.4 Meta-analysis

#### 3.4.1 Scr

Thirteen studies reported changes in Scr after UCMSC-Exo treatment. A random-effects model (I^2^ = 83.8%, P < 0.0001) showed a significant reduction in Scr compared with controls (SMD = −5.60, 95% CI −7.61 to −3.60; P < 0.05) (Supplementary Figure S1).

**FIGURE 3:**
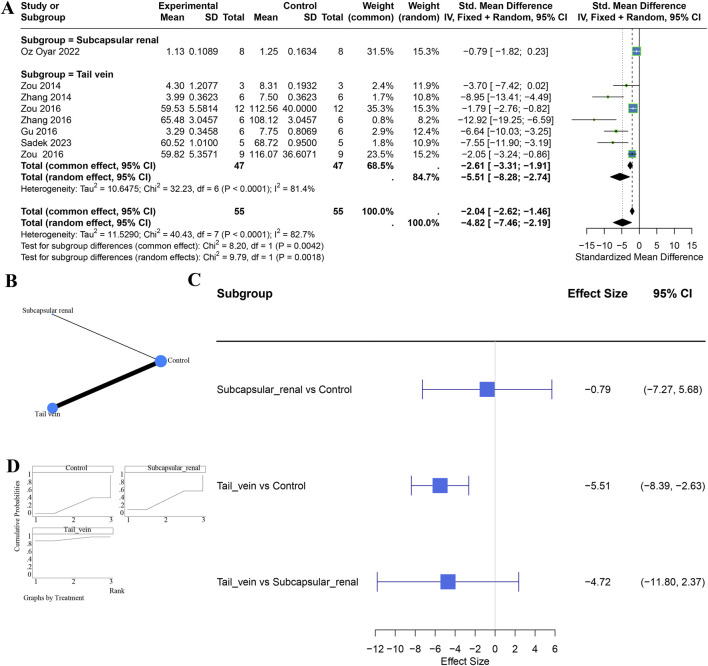
Comparative effects of different routes of administration on serum creatinine reduction at a fixed 100 μg dose. Panels: **(A)** subgroup analysis; **(B)** evidence network; **(C)** SUCRA-based ranking; **(D)** network meta-analysis forest plot.

We further examined the effect of delivery route at a fixed dose of 100 μg. Eight studies were included in this subgroup analysis (renal subcapsular injection, n = 1; tail-vein injection, n = 7). Under a random-effects model, tail-vein injection significantly reduced Scr (SMD = −5.51, 95% CI −8.28 to −2.74), whereas renal subcapsular injection did not (SMD = −0.79, 95% CI −1.82 to 0.23). The test for subgroup differences was significant (χ^2^ = 9.79, P = 0.0018), suggesting an advantage for tail-vein delivery; heterogeneity within subgroups remained high (I^2^ = 82.7%) (Figure 3A). Network meta-analysis corroborated that, versus control, tail-vein injection was superior to renal subcapsular injection, and ranking analyses indicated that at 100 μg the tail-vein route was most likely to be optimal (Figures 3B–D). Consistency was assessed using the node-splitting method, and the results showed no statistically significant difference between direct and indirect comparisons (P > 0.05), indicating good consistency. Data for other doses were limited and were not analyzed further.

To clarify dose effects within the tail-vein route, we performed subgroup and network meta-analyses across 9 studies: 100 μg (7 studies), 30 μg (1 study), and 250 μg (1 study). All doses significantly lowered Scr in random-effects models, and there was no significant difference between dose groups (χ^2^ = 0.10, df = 2, P = 0.9489). Network meta-analysis detected no significant pairwise differences; ranking suggested 100 μg had the highest probability of being the best dose for Scr reduction (Supplementary Figure S2). Because the network structure was star-shaped (all interventions connected through a common control group, with no direct comparisons between interventions), closed loops could not be formed, and thus the loop-specific approach was not applicable.

Within the renal subcapsular route (4 studies: 100 μg [n = 1], 200 μg [n = 2], 400 μg [n = 1]), effects varied. The 200 μg and 400 μg groups tended to reduce Scr, with the 400 μg dose achieving statistical significance, whereas 100 μg did not. Subgroup differences were significant (χ^2^ = 14.47, df = 2, P = 0.0007). Network meta-analysis indicated that 200 μg may outperform 100 μg, while comparisons involving 400 μg versus 100 or 200 μg were not statistically significant. Ranking suggested that 200 μg and 400 μg had higher probabilities of being optimal. Overall, a dose-dependent trend was observed for the renal subcapsular route, with higher doses (200–400 μg) appearing more effective than 100 μg (Supplementary Figure S3). As this network was also star-shaped, loop-specific testing was not applicable.

#### 3.4.2 Renal injury score

Nine studies reported histological injury scores. Using a random-effects model (I^2^ = 65.6%, P = 0.0031), UCMSC-Exo significantly reduced injury scores compared with controls (SMD = −3.62, 95% CI −4.91 to −2.34; P < 0.05) (Supplementary Figure S4).

**FIGURE 4:**
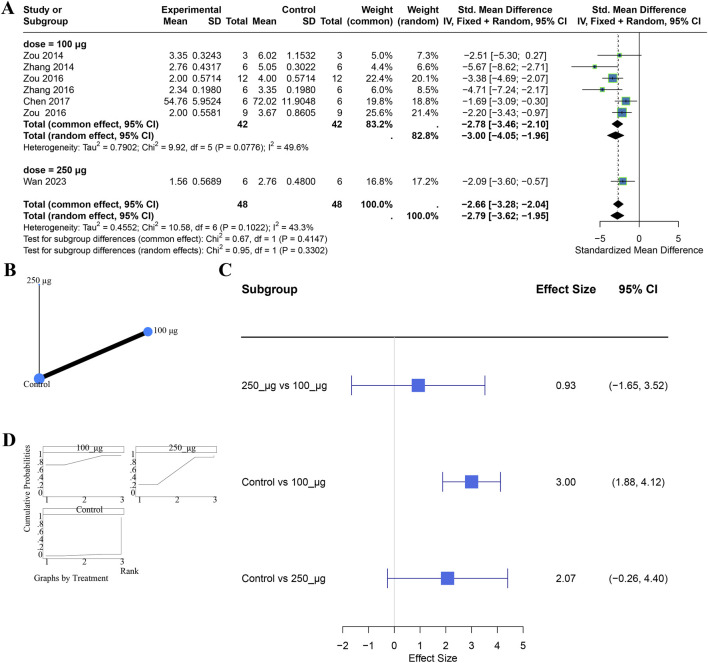
Comparative effects of tail-vein administration at different doses on renal injury scores. Panels: **(A)** subgroup analysis; **(B)** evidence network; **(C)** SUCRA-based ranking; **(D)** network meta-analysis forest plot.

We then assessed dose effects for tail-vein delivery. Seven studies contributed data (100 μg, n = 6; 250 μg, n = 1). Both 100 μg and 250 μg significantly reduced injury scores. The test for subgroup differences was not significant (χ^2^ = 0.95, df = 1, P = 0.3302), with modest heterogeneity (I^2^ = 43.3%) (Figure 4A). Network meta-analysis likewise found no significant difference between the two doses; ranking favored 100 μg as the most likely optimal dose (Figures 4B–D). Consistency was assessed using the node-splitting method, and the results showed no statistically significant difference between direct and indirect comparisons (P > 0.05). Evidence for the renal subcapsular route was insufficient to explore dose–response patterns or to compare routes at specific doses for this endpoint.

#### 3.4.3 TUNEL positive cell

Eight studies assessed apoptosis using TUNEL-positive cell counts. A random-effects model (I^2^ = 78.6%, P < 0.0001) showed a significant reduction with UCMSC-Exo compared with controls (SMD = −4.35, 95% CI −6.06 to −2.64; P < 0.05) (Supplementary Figure S5).

**FIGURE 5:**
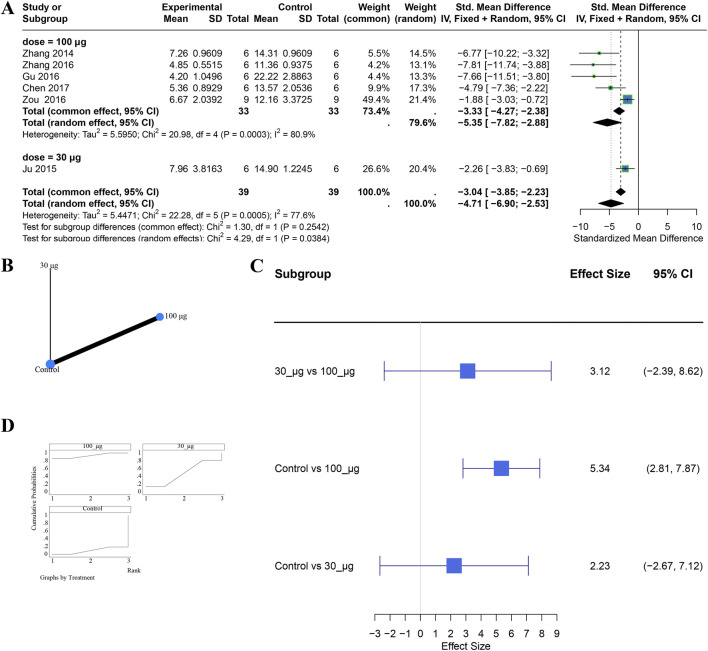
Comparative effects of tail-vein administration at different doses on renal apoptosis. Panels: **(A)** subgroup analysis; **(B)** evidence network; **(C)** SUCRA-based ranking; **(D)** network meta-analysis forest plot.

Within the tail-vein route (6 studies: 100 μg [n = 5] and 30 μg [n = 1]), both doses significantly reduced TUNEL-positive cells. The test for subgroup differences was significant (P = 0.0384) (Figure 5A). However, network meta-analysis found no statistically significant differences in direct or indirect comparisons between 100 μg and 30 μg; ranking analyses indicated that 100 μg had the highest probability of being the optimal dose (Figures 5B–D). Consistency was assessed using the node-splitting method, and the results showed no statistically significant difference between direct and indirect comparisons (P > 0.05). Data were insufficient to evaluate dose effects within the renal subcapsular route or to compare routes at specific doses for apoptosis outcomes.

#### 3.4.4 BUN

A total of 13 studies reported changes in blood urea nitrogen levels following UCMSC-Exo treatment. Conventional meta-analysis using a random-effects model (I^2^ = 85.8%, P < 0.0001) showed that, compared with controls, UCMSC-Exo intervention significantly reduced BUN levels in rat AKI models (SMD = −6.33, 95% CI: −8.91 to −3.75), and this difference was statistically significant (P < 0.05) (Supplementary Figure S6).

We further assessed the impact of different administration routes on BUN at a fixed dose of 100 μg. Eight studies were included in this subgroup analysis, comprising 3 with subcapsular renal injection and 5 with tail vein injection. Random-effects modeling indicated that, at a dose of 100 μg, tail vein injection significantly reduced BUN levels (SMD = −5.66, 95% CI: −8.89 to −2.43), whereas subcapsular renal injection did not show a statistically significant effect (SMD = −2.81, 95% CI: −7.24 to 0.62), suggesting that tail vein injection may be more advantageous for lowering BUN (Supplementary Figure S7A). We then used network meta-analysis to compare the direct and indirect evidence for the two routes. The results showed no statistically significant difference in efficacy between tail vein and subcapsular renal injection. However, ranking probability analysis indicated that, at a dose of 100 μg, tail vein injection was the most likely to be the optimal route for reducing BUN (Supplementary Figures S7B–D). Consistency was assessed using the node-splitting method, and the results showed no statistically significant difference between direct and indirect comparisons (P > 0.05). Taken together, under the fixed dose of 100 μg, tail vein injection appeared to confer a more pronounced improvement in BUN in AKI rats than subcapsular renal injection. It should be noted that data for other doses were limited and were therefore not analyzed in depth.

To clarify dose-related differences in the efficacy of BUN reduction via the tail vein route, we conducted additional subgroup and network meta-analyses. Nine studies were included and categorized by UCMSC-Exo dose into five subgroups: 100 μg (5 studies), 30 μg (1 study), 200 μg (1 study), 250 μg (1 study), and 400 μg (1 study). Random-effects modeling showed that all dose groups significantly reduced BUN levels. Tests for between-subgroup differences indicated statistically significant heterogeneity in efficacy among the dose groups (random-effects model: Chi^2^ = 22.04, P = 0.0002). Network meta-analysis was then used to indirectly compare the different doses. The results showed no statistically significant pairwise differences in efficacy between any two dose groups. Ranking probability analysis suggested that, among tail vein injections, a dose of 100 μg had the highest probability of being associated with the most favorable trend in BUN reduction (Supplementary Figure S8). Because the network structure was star-shaped, closed loops could not be formed, and thus the loop-specific approach was not applicable.

We also evaluated the impact of different UCMSC-Exo doses on BUN when administered via subcapsular renal injection. Four studies were included and divided into two subgroups according to dose: 100 μg (3 studies) and 200 μg (1 study). Random-effects modeling indicated variability in efficacy between dose groups. The 200 μg dose showed a trend toward reducing BUN, whereas the 100 μg dose did not demonstrate a significant effect. Tests for between-subgroup differences suggested statistically significant heterogeneity in efficacy across doses (random-effects model: Chi^2^ = 4.74, P = 0.0294). Network meta -analysis was used for indirect comparisons between doses and revealed no statistically significant differences among the dose groups. Ranking probability analysis indicated that, for subcapsular renal injection, a dose of 200 μg had a higher probability of being the optimal dose. Overall, these findings suggest a dose-dependent trend for UCMSC-Exo administered via subcapsular renal injection, with higher doses potentially being more effective than lower doses in improving BUN (Supplementary Figure S9). As this network was also star-shaped, loop-specific testing was not applicable.

#### 3.4.5 Publication bias

Publication bias assessment for Scr revealed asymmetry upon visual inspection of the funnel plot (Supplementary Figure S10A). Both Begg’s test (P < 0.05) and Egger’s test (P < 0.05) indicated the presence of statistically significant publication bias (Supplementary Figures S10B,C). Further trim-and-fill analysis estimated six missing studies, and the adjusted pooled effect size changed from the original SMD = −5.60 (95% CI: −7.61 to −3.60) to SMD = −2.59 (95% CI: −4.51 to −0.60) (Supplementary Figure S10D). Publication bias assessment for BUN also suggested the possibility of publication bias in the available studies. The estimated number of missing studies was six, and the adjusted pooled effect size changed from the original SMD = −6.33 (95% CI: −8.91 to −3.75) to SMD = −2.54 (95% CI: −3.46 to −1.74) (Supplementary Figure S11). Although the adjusted effect sizes were attenuated, they remained statistically significant, suggesting that the efficacy of UCMSC-Exos in reducing serum creatinine is robust, but the true effect size may be overestimated by the available evidence, and the impact of publication bias should be considered when interpreting the results.

## 4 Discussion

By combining conventional and network meta-analytic approaches, this study comprehensively evaluated the efficacy of UCMSC-Exos in rat models of AKI and systematically explored the optimal administration strategy. Overall, the analyses showed that UCMSC-Exo treatment significantly reduced serum creatinine and blood urea nitrogen levels, improved renal histopathological injury scores, and inhibited cell apoptosis. The consistent statistical significance observed across multiple key endpoints provides robust evidence to support the potential of UCMSC-Exos as a novel cell-free therapeutic strategy in AKI regenerative medicine.

UCMSC-Exos exerted marked benefits on serum creatinine, blood urea nitrogen, renal injury scores, and TUNEL-positive cell counts in AKI, suggesting a multitarget, multipathway therapeutic profile. This finding aligns well with current mechanistic insights into MSC-derived exosomes. As a naturally occurring nanoscale mediator of intercellular communication, UCMSC-Exos are enriched in bioactive cargos, including microRNAs, mRNAs, proteins, and lipids, which can simultaneously modulate multiple pathophysiological processes (Tang et al., 2021; Wang et al., 2025). At the molecular level, previous studies have demonstrated that UCMSC-Exos can deliver miR--5p to target TEAD1, thereby inhibiting apoptosis and pyroptosis of tubular epithelial cells and conferring renoprotective effects (Li et al., 2025). Our meta-analysis showed a significant reduction in TUNEL-positive cells following UCMSC-Exo treatment, providing macro-level support for this anti-apoptotic mechanism. In addition, UCMSC-Exos can deliver miR-146b to modulate inflammatory responses, suppress excessive activation of the NF-κB signaling pathway, and attenuate inflammatory injury in the kidney (Zhang et al., 2020). The pronounced decreases in serum creatinine and blood urea nitrogen and the improvements in renal histological injury scores observed in this study likely reflect the integrated contribution of these anti-inflammatory, anti-apoptotic, and anti-fibrotic mechanisms.

An important finding of this study is that the therapeutic efficacy of UCMSC-Exos differs markedly between administration routes, and that this difference is closely linked to the administered dose. At a fixed dose of 100 μg, tail vein injection was significantly superior to subcapsular renal injection in improving serum creatinine, and showed a similar trend toward superiority in reducing blood urea nitrogen. However, this does not imply that subcapsular renal injection is intrinsically ineffective; rather, it suggests that the commonly used dose of 100 μg may not reach the threshold required for therapeutic efficacy with this route. Indeed, our dose–response analyses further demonstrated a clear dose dependence for subcapsular renal injection. Doses of 200 μg and 400 μg both showed a trend toward reducing serum creatinine, with 400 μg achieving statistical significance; for BUN, 200 μg was also significantly more effective than 100 μg. These observations are consistent with reports that high-dose subcapsular renal injection can improve renal function and help explain the heterogeneous efficacy reported for this route in previous studies, where dose differences may have been a key determinant. A more in-depth examination of the underlying mechanisms suggests that these findings are closely related to the fundamentally distinct pharmacokinetic properties, biodistribution patterns, and impacts on systemic versus local pathophysiology of the two administration routes. From a pharmacokinetic and bioavailability perspective, tail vein injection, as a systemic delivery route, allows exosomes entering the circulation to rapidly distribute throughout the body. Although a proportion will inevitably be sequestered and cleared by organs rich in mononuclear phagocyte systems, such as the liver and spleen, this “first-pass effect” may also endow exosomes with the capacity to modulate systemic immune status (Elliott and He, 2021; Tavasolian et al., 2021). More importantly, circulating exosomes can repeatedly and continuously perfuse the kidney via the renal arterial blood flow, reaching key targets such as the glomeruli and peritubular capillary network around the renal tubules, thereby exerting broad and balanced effects on the functional units of the entire kidney (Zhang et al., 2025; Quaglia et al., 2022; Zhang et al., 2023). By contrast, subcapsular renal injection is intended as a local delivery strategy to achieve high exosome concentrations within the kidney. However, our findings indicate suboptimal efficacy, which may be attributable to the renal capsule, a dense connective tissue layer that can impede effective diffusion of exosomes into the renal parenchyma. As a result, exosomes may remain largely confined to the injection site, failing to distribute evenly throughout the cortex and medulla, and particularly struggling to reach the tubular epithelial cells in the regions of most severe injury (Grange and Bussolati, 2022). In addition, local immune cells at the injection site, such as macrophages, may rapidly engulf and clear the high local concentration of exosomes, shortening their local half-life in the kidney and diminishing sustained therapeutic effects (Chen et al., 2024; Choi et al., 2021). These barrier effects help explain why subcapsular renal injection requires higher doses to achieve efficacy comparable to intravenous administration, and why only when the dose is sufficiently increased to overcome local diffusion barriers and phagocytic clearance can an effective therapeutic concentration be established in the target tissue.

From the perspective of immune modulation, AKI is not merely an isolated renal event; its onset and progression are often accompanied by, or even driven by, systemic inflammatory response syndrome (SIRS), which in turn exacerbates AKI and creates a vicious cycle (Ostermann et al., 2025). After tail vein injection, UCMSC-Exos enter the bloodstream and can interact extensively with circulating immune cells such as monocytes, neutrophils, and lymphocytes, modulating their activation and differentiation and suppressing excessive systemic inflammation, thereby indirectly alleviating renal inflammatory injury (Quaglia et al., 2020; Ding et al., 2024). This systemic immunomodulatory effect is difficult to achieve with subcapsular renal injection. Although the latter may establish a high-concentration immunoregulatory microenvironment within the kidney, its capacity to modulate an established systemic inflammatory “storm” remains relatively limited.

Our findings on the dose–response relationships within each administration route indicate a clear divergence between tail vein and subcapsular renal injection. For tail vein injection, there was no significant difference in efficacy among the 30 μg, 100 μg, and 250 μg doses, suggesting a “nonlinear” or “plateau” effect. This pattern was consistently observed for both Scr and BUN. These results imply that the action of UCMSC-Exos may follow a “receptor saturation” or “threshold” model: once the intravenous dose reaches a certain level (e.g., 30 μg), the engagement of specific receptors on target cells (such as immune cells and renal vascular endothelial cells) or the activation of downstream signaling pathways may already be saturated or at a maximal threshold. Further dose escalation does not proportionally enhance efficacy and may instead lead to excess exosomes being cleared by the mononuclear phagocyte system, resulting in unnecessary waste (Oz Oyar et al., 2022). In addition, this phenomenon may relate to an exosome-mediated “paracrine/endocrine” amplification effect, whereby a relatively low dose is sufficient to trigger a cascade of reparative responses by activating endogenous repair mechanisms.

By contrast, for subcapsular renal injection, we observed a clear dose-dependent trend. This further underscores the bioavailability challenges associated with local delivery. At the lower dose of 100 μg, the aforementioned diffusion barriers and rapid local clearance may prevent UCMSC-Exos from achieving therapeutically effective concentrations within the target tissue. Only when the dose is increased to 200 μg or even 400 μg can these barriers be overcome, establishing a sufficient concentration gradient to drive exosome penetration into deeper renal tissue and maintaining an effective local concentration for a longer duration, thereby exerting therapeutic effects. This illustrates the strong dose dependence of local administration, in which higher doses are required to achieve adequate tissue exposure.

Taken together, tail vein injection has the advantage of more effectively enabling systemic immune modulation and uniform distribution throughout the kidney, with a likely saturable mechanism of action, making 100 μg an economically efficient preferred dose. In contrast, subcapsular renal injection is constrained by local barriers and rapid clearance and therefore requires higher doses to achieve meaningful efficacy. These findings have important implications for clinical practice: if an intravenous route is selected, a dose of 100 μg may represent the most cost-effective option; if a local injection route is chosen, higher doses should be considered to attain the therapeutic effect.

When interpreting the above findings, the limitations of this study must be fully considered. First, although 14 studies were included, the number of studies in some subgroup analyses remained small. For example, only 4 studies compared different doses via the subcapsular renal injection route, which limited statistical power and increased the risk of false-negative results. In addition, some comparisons (e.g., 30 μg vs. 250 μg) were based on only 1–2 studies, and the reliability of these findings warrants further validation. Due to the extremely small number of studies within certain subgroups (e.g., only 1 study in the 100 μg subcapsular renal injection group and 1 study in the 30 μg tail vein injection group), sensitivity analyses by excluding individual studies would not have yielded meaningful pooled effect sizes; therefore, conventional sensitivity analysis was not performed in this study. We comprehensively evaluated the robustness of the findings through cross-validation between subgroup analyses and network meta-analyses, as well as consistency across multiple outcome measures. Second, it must be acknowledged that the original studies included in this analysis generally suffered from small sample sizes and suboptimal methodological reporting, which directly lowers the overall strength of the current evidence base. Paradoxically, these limitations in the primary data highlight the core value of the present work: by systematically integrating limited and heterogeneous evidence, this study not only identifies, for the first time, the potential optimal strategy for UCMSC-Exo therapy in AKI, but also, and more importantly, quantitatively exposes key weaknesses in the current preclinical research landscape (such as non-uniform modeling methods, sex bias, and inadequate sample sizes). Thus, beyond providing comparative efficacy data, this study has an “early warning” function, offering clear guidance for designing more rigorous, larger-scale animal experiments and thereby promoting the field toward higher-quality and more translatable research. Third, although we used random-effects models to account for between-study heterogeneity, I^2^ values remained high in some analyses. This heterogeneity may stem from multiple sources, including rat strain, age, sex, AKI model type, exosome preparation methods, and follow-up duration. While subgroup analyses were performed, they did not fully explain the observed heterogeneity. Fourth, the assessment of renal fibrosis in this study was clearly inadequate. Tubulointerstitial fibrosis is a key step in the progression from AKI to chronic kidney disease, yet only 4 of the 14 included studies reported fibrosis-related endpoints. Moreover, substantial heterogeneity in assessment methods and time points precluded meta-analysis. As a result, this study cannot elucidate the impact of UCMSC-Exos on long-term fibrotic outcomes. Future primary studies should extend follow-up periods and use standardized methods to systematically evaluate fibrosis-related markers. Fifth, regarding publication bias, significant publication bias was detected in the analyses of serum creatinine and BUN. After correction using the trim-and-fill method, the effect sizes were attenuated, suggesting that publication bias may have overestimated the true efficacy of UCMSC-Exos. Potential reasons include that studies with positive results are more likely to be accepted and published, while negative results may be shelved or rejected; the diversity in exosome preparation and administration protocols may lead some investigators to preferentially report outcomes from more optimal regimens; and the generally small sample sizes (6–24 per group) of the included studies may be associated with larger effect size estimates. Of particular note, inconsistency in AKI modeling methods is one of the most critical limitations of this study. Different AKI induction methods involve distinct pathophysiological mechanisms. Cisplatin-induced nephrotoxicity primarily causes tubular epithelial cell apoptosis through mitochondrial dysfunction and DNA damage, whereas ischemia–reperfusion injury involves multiple mechanisms including oxidative stress, inflammatory cascades, endothelial dysfunction, and microcirculatory disturbances (Fang et al., 2021; Tang et al., 2023; Kwiatkowska et al., 2023). Even within ischemia–reperfusion models, important differences exist among unilateral, bilateral, and unilateral models combined with contralateral nephrectomy. Specifically, in unilateral ischemia–reperfusion models (4 studies in this review), the presence of a contralateral healthy kidney exerts compensatory filtration that significantly affects the interpretation of systemic renal function indicators such as Scr and BUN, potentially leading to underestimation of the actual protective effect of UCMSC-Exos on the injured kidney. In contrast, bilateral ischemia models (2 studies) and unilateral ischemia combined with contralateral nephrectomy models (4 studies) avoid this compensatory effect and more accurately reflect the direct action of exosomes on the injured kidney. Therefore, future studies employing unilateral ischemia models should incorporate more sensitive and kidney-specific assessment indicators, such as kidney injury molecule-1 (KIM-1), neutrophil gelatinase-associated lipocalin (NGAL), tubular injury markers (e.g., β2-microglobulin, N-acetyl-β-D-glucosaminidase), as well as quantitative histological scoring (e.g., percentage of tubular necrosis area, grading of interstitial inflammatory infiltration), to more comprehensively and accurately evaluate injury and repair in the affected kidney (Hukriede et al., 2022; Zhang et al., 2024). Additionally, among the included studies, ischemia times ranged from 40 to 60 min, and reperfusion times were not reported; these variations directly affect the severity of injury and the assessment window for the repair phase. However, because the primary stratification variables in this study were administration route and dose, further stratification by modeling method would have resulted in too few studies per subgroup to permit meaningful meta-analysis or meta-regression. Consequently, our analysis could not quantitatively assess the potential moderating effect of modeling methods on treatment efficacy. This limitation restricts the generalizability of our findings. We cannot determine whether the “optimal strategy” of 100 μg via tail vein injection is equally effective across all AKI subtypes. Moreover, differences in sex hormone levels have been shown to influence susceptibility to AKI and repair processes, and may also modulate the efficacy of stem cells and their derivatives (Lima-Posada and Bobadilla, 2021; Neugarten et al., 2018). However, due to the inconsistent use of animal sexes across included studies and the limited number of studies, we were unable to conduct subgroup analyses or meta-regression to quantitatively assess the effect of sex on treatment outcomes. Collectively, modeling methods and animal sex represent the two most important confounding factors that could not be quantitatively analyzed in this study, necessitating caution when interpreting our conclusions. In addition, publication bias testing revealed significant publication bias in the serum creatinine analysis. Although the adjusted effect size remained statistically significant, its magnitude was notably smaller than the original estimate. This suggests that the true efficacy of UCMSC-Exos may be overestimated in this study, warranting caution in result interpretation. Finally, the methodological quality of the included studies was variable. All studies were rated as “unclear risk” for domains such as “sequence generation,” “allocation concealment,” “random housing,” and “blinding of caregivers/investigators,” largely due to inadequate reporting in the original publications. Only a subset of studies reported key information such as baseline comparability and blinding of outcome assessors. These methodological shortcomings may affect the internal validity of the findings.

## 5 Conclusion

In summary, our findings demonstrate that UC-MSC-Exos exert significant therapeutic effects in rat models of AKI by improving renal function, attenuating tissue injury, and suppressing cell apoptosis. More importantly, we identified clear differences in efficacy between administration routes: tail vein injection was significantly superior to subcapsular renal injection in improving serum creatinine, and the optimal dose varied by route, with 100 μg emerging as the optimal dose for tail vein injection and a dose-dependent pattern observed for subcapsular renal injection. These results provide important evidence-based support for the clinical translation of UC-MSC-Exos and lay the groundwork for developing individualized treatment strategies.

However, it must be emphasized that substantial heterogeneity existed among the included studies in terms of AKI modeling methods (including induction approach, ischemia time, and reperfusion time) and animal sex, and we were unable to quantitatively assess the moderating effects of these factors on treatment efficacy. Therefore, the above conclusions should be regarded as exploratory insights based on the current evidence rather than definitive practice guidelines. This study also has limitations, including limited sample size, high between-study heterogeneity, and potential publication bias. Future work should validate and refine our findings through more rigorously designed and better reported primary studies, including detailed descriptions and standardization of modeling parameters and procedures, as well as balanced inclusion of both sexes.
