# Supplementary material for: Optimal strategies for umbilical cord mesenchymal stem cell–derived exosomes in acute kidney injury: a network meta-analysis in rat models
Source: Front Cell Dev Biol. 2026 Apr 28;14:1794218. doi: 10.3389/fcell.2026.1794218 (PMC13161154; doi:10.3389/fcell.2026.1794218)
Supplement: Supplementary file 1 [file DataSheet1.docx]

**Optimal Strategies for Umbilical Cord Mesenchymal Stem Cell–Derived Exosomes in Acute Kidney Injury: A Network Meta-analysis in Rat Models**

Supplementary Table 1. Search strategy.

| 1. WOS  (TS=("acute kidney injury" OR "acute renal injury" OR "acute kidney failure" OR "acute renal failure" OR "acute renal insufficiency" OR "acute kidney insufficiency")) AND TS=("exosome*" OR "exosomes" OR "exosomal" OR "extracellular vesicle" OR "extracellular vesicles" OR "extracellular particle" OR "extracellular particles" OR "microvesicle" OR "microvesicles" OR "Shedding Microvesicle" OR "Shedding Microvesicles" OR "Secretory Vesicle" OR "Secretory Vesicles" OR "Cell-Derived Microparticle" OR "Cell-Derived Microparticles" OR "microbubble" OR "microbubbles" OR "apoptotic body" OR "apoptosis bodies")  2. PubMed  #1: "Exosomes"[MeSH Terms] OR "Extracellular Vesicles"[MeSH Major Topic] OR "Cell-Derived Microparticles"[MeSH Major Topic] OR "Microbubbles"[MeSH Major Topic]  #2: "exosome*"[Title/Abstract] OR "exosomes"[Title/Abstract] OR "exosomal"[Title/Abstract] OR "extracellular vesicle"[Title/Abstract] OR "extracellular vesicles"[Title/Abstract] OR "extracellular particle"[Title/Abstract] OR "extracellular particles"[Title/Abstract] OR "microvesicle"[Title/Abstract] OR "microvesicles"[Title/Abstract] OR "Shedding Microvesicle"[Title/Abstract] OR "Shedding Microvesicles"[Title/Abstract] OR "Secretory Vesicle"[Title/Abstract] OR "Secretory Vesicles"[Title/Abstract] OR "Cell-Derived Microparticle"[Title/Abstract] OR "Cell-Derived Microparticles"[Title/Abstract] OR "microbubble"[Title/Abstract] OR "microbubbles"[Title/Abstract] OR "apoptotic body"[Title/Abstract] OR "apoptosis bodies"[Title/Abstract]  #3: #1 OR #2  #4: "Acute Kidney Injury"[MeSH Major Topic]  #5: "acute kidney injury"[Title/Abstract] OR "acute renal injury"[Title/Abstract] OR "acute kidney failure"[Title/Abstract] OR "acute renal failure"[Title/Abstract] OR "acute renal insufficiency"[Title/Abstract] OR "acute kidney insufficiency"[Title/Abstract]  #6: #4 OR #5  #7: #3 AND #6  3. Scopus  TITLE-ABS-KEY ( "acute kidney injury" OR "acute renal injury" OR "acute kidney failure" OR "acute renal failure" OR "acute renal insufficiency" OR "acute kidney insufficiency" ) AND TITLE-ABS-KEY ( "exosome*" OR "exosomes" OR "exosomal" OR "extracellular vesicle" OR "extracellular vesicles" OR "extracellular particle" OR "extracellular particles" OR "microvesicle" OR "microvesicles" OR "Shedding Microvesicle" OR "Shedding Microvesicles" OR "Secretory Vesicle" OR "Secretory Vesicles" OR "Cell-Derived Microparticle" OR "Cell-Derived Microparticles" OR "microbubble" OR "microbubbles" OR "apoptotic body" OR "apoptosis bodies" ) AND ( LIMIT-TO ( DOCTYPE , "ar" ) OR LIMIT-TO ( DOCTYPE , "re" ) )  4. Embase  #1: exosome*:ti,ab,kw OR exosomes:ti,ab,kw OR exosomal:ti,ab,kw OR 'extracellular vesicle':ti,ab,kw OR 'extracellular vesicles':ti,ab,kw OR 'extracellular particle':ti,ab,kw OR 'extracellular particles':ti,ab,kw OR 'microvesicle':ti,ab,kw OR 'microvesicles':ti,ab,kw OR 'shedding microvesicle':ti,ab,kw OR 'shedding microvesicles':ti,ab,kw OR 'secretory vesicle':ti,ab,kw OR 'secretory vesicles':ti,ab,kw OR 'cell-derived microparticle':ti,ab,kw OR 'cell-derived microparticles':ti,ab,kw OR 'microbubble':ti,ab,kw OR 'microbubbles':ti,ab,kw OR 'apoptotic body':ti,ab,kw OR 'apoptosis bodies':ti,ab,kw  #2: 'exosome'/exp  #3: 'exosomes'/exp  #4: 'extracellular vesicle'/exp  #5: 'microvesicle'/exp  #6: 'secretory vesicle'/exp  #7: 'cell-derived microparticle'/exp  #8: 'microbubble'/exp  #9: 'apoptotic body'/exp  #10: #1 OR #2 OR #3 OR #4 OR #5 OR #6 OR #7 OR #8 OR #9  #11: 'acute kidney injury':ab,ti OR 'acute renal injury':ab,ti OR 'acute kidney failure':ab,ti OR 'acute renal failure':ab,ti OR 'acute renal insufficiency':ab,ti OR 'acute kidney insufficiency':ab,ti  #12: 'acute kidney injury'/exp  #13: 'acute renal injury'/exp  #14: #11 OR #12 OR #13  #15: #10 AND #14 ('article'/it OR 'clinical trial'/it OR 'review'/it) |
| --- |

Supplementary Table 2. Characteristics of the included studies.

| **No.** | **Author** | **Year** | **Country** | **Research type** | **Animal Characteristics** | | | | **Sample Size** | **Modeling Method** | **Exosome** | | **Transplant Timing/Frequency** | **Transplant Route** | **Transplant Dose** | Control Group |
| --- | --- | --- | --- | --- | --- | --- | --- | --- | --- | --- | --- | --- | --- | --- | --- | --- |
|  |  |  |  |  | **Species** | **Gender** | **Weight** | **Age** | **Experimental/Control Group** |  | **Type** | **Source** |  |  |  |  |
| 1 | Oz Oyar | 2022 | Turkey | RCT | Wistar rats | Male | / | / | 8/8 | Cisplatin-induced acute kidney injury (AKI) | HucMSC-Exo | Collection of hUCMSC-conditioned medium | Single transplantation 24 hours after cisplatin injection | Subcapsular renal injection | 100 μg | Physiological saline |
| 2 | Zou | 2016 | China | RCT | SD rats | Male | 180–200 g | / | 12/12 | Right nephrectomy followed by clamping of the left renal pedicle for 45 minutes, with subsequent reperfusion | HucMSC-Exo | Collection of hUCMSC-conditioned medium | Single administration immediately after reperfusion | Tail vein | 100 μg | Culture medium (without Exo) |
| 3 | Wang | 2017 | China | RCT | SD rats | Female | 210–250 g | 6–8 weeks | 6/6 | Single intraperitoneal injection of cisplatin (5 mg/kg) | HucMSC-Exo | Collection of hUCMSC-conditioned medium | Single pretreatment 0.5 hours before cisplatin injection | Subcapsular renal injection | 200 μg | PBS (Phosphate-buffered saline) |
| 4 | Zhang | 2016 | China | RCT | SD rats | Male | 180–200 g | 8 weeks | 6/6 | Clamping of the left renal artery for 45 minutes after right nephrectomy, followed by reperfusion | HucMSC-Exo | Collection of hUCMSC-conditioned medium | Single transplantation immediately after reperfusion | Tail vein | 100 μg | Equal volume (1 mL) of medium M199 |
| 5 | Zou | 2016 | China | RCT | SD rats | Male | 180-200 g | 8 weeks | 18/18 | After right nephrectomy, the left renal artery was clamped with an atraumatic vascular clamp for 45 minutes, followed by restoration of blood flow | HucMSC-Exo | Collection of hUCMSC-conditioned medium | Single intravenous administration immediately after reperfusion | Tail vein | 100 μg | Equivalent culture medium M199 |
| 6 | Jia | 2018 | China | RCT | SD rats | Female | 220 ± 20 g | / | 6/6 | Single intraperitoneal injection of cisplatin (5 mg/kg) | HucMSC-Exo | Collection of hUCMSC-conditioned medium | Single administration of exosomes 24 hours before cisplatin injection | Subcapsular renal injection | 200 μg | PBS (Phosphate-buffered saline) |
| 7 | Chen | 2017 | China | RCT | SD rats | Male | 180-200 g | / | 24/24 | Clamping of the left renal artery for 60 minutes, followed by reperfusion | HucMSC-Exo | Collection of hUCMSC-conditioned medium | Single injection immediately after reperfusion | Tail vein | 100 μg | DMEM (Dulbecco's Modified Eagle Medium) |
| 8 | Zou | 2014 | China | RCT | SD rats | Male | 180-200 g | / | 6/6 | Unilateral left renal ischemia for 60 minutes followed by reperfusion | HucMSC-Exo | Collection of hUCMSC-conditioned medium | Single transplantation immediately after reperfusion | Tail vein | 100 μg | Equivalent culture medium M199 |
| 9 | Sadek | 2023 | Egypt | RCT | Albino rats | Male | 180-200 g | Adult | 10/10 | Bilateral renal pedicles were ligated for 40 minutes to induce renal ischemia, followed by release to allow reperfusion | HucMSC-Exo | Collection of hUCMSC-conditioned medium | Single injection immediately after reperfusion | Tail vein | 100 μg | Physiological saline |
| 10 | Wan | 2023 | China | RCT | SD rats | Male | 190–220 g | / | 6/6 | Bilateral renal vessels were clamped with atraumatic arterial clamps for 40 minutes, followed by reperfusion | HucMSC-Exo | Collection of hUCMSC-conditioned medium | Single injection immediately after reperfusion | Tail vein | 250 µg | Control blank |
| 11 | Zhou | 2013 | China | RCT | SD rats | Female | 220 ± 20 g | / | 6/6 | Single intraperitoneal injection of cisplatin (6 mg/kg) | HucMSC-Exo | Collection of hUCMSC-conditioned medium | Single transplantation 24 hours after cisplatin injection | Subcapsular renal injection | 400 μg | PBS (Phosphate-buffered saline) |
| 12 | Zhang | 2014 | China | RCT | SD rats | Male | 180–200 g | / | 6/6 | The left renal pedicle was clamped with an atraumatic vascular clamp for 60 minutes, followed by restoration of blood flow | HucMSC-Exo | Collection of hUCMSC-conditioned medium | Single injection immediately after model establishment | Tail vein | 100 μg | Equivalent culture medium M199 |
| 13 | Ju | 2015 | China | RCT | SD rats | Male | 180–210 g | Adult | 24/24 | Left renal ischemia for 60 minutes | HucMSC-Exo | Collection of hUCMSC-conditioned medium | Single transplantation immediately after reperfusion | Tail vein | 30 μg | Equivalent culture medium M199 |
| 14 | Gu | 2016 | China | RCT | SD rats | Male | 180-200 g | / | 6/6 | Right nephrectomy combined with clamping of the left renal pedicle for 45 minutes | HucMSC-Exo | Collection of hUCMSC-conditioned medium | Single transplantation immediately after reperfusion | Tail vein | 100 μg | Equivalent culture medium M199 |


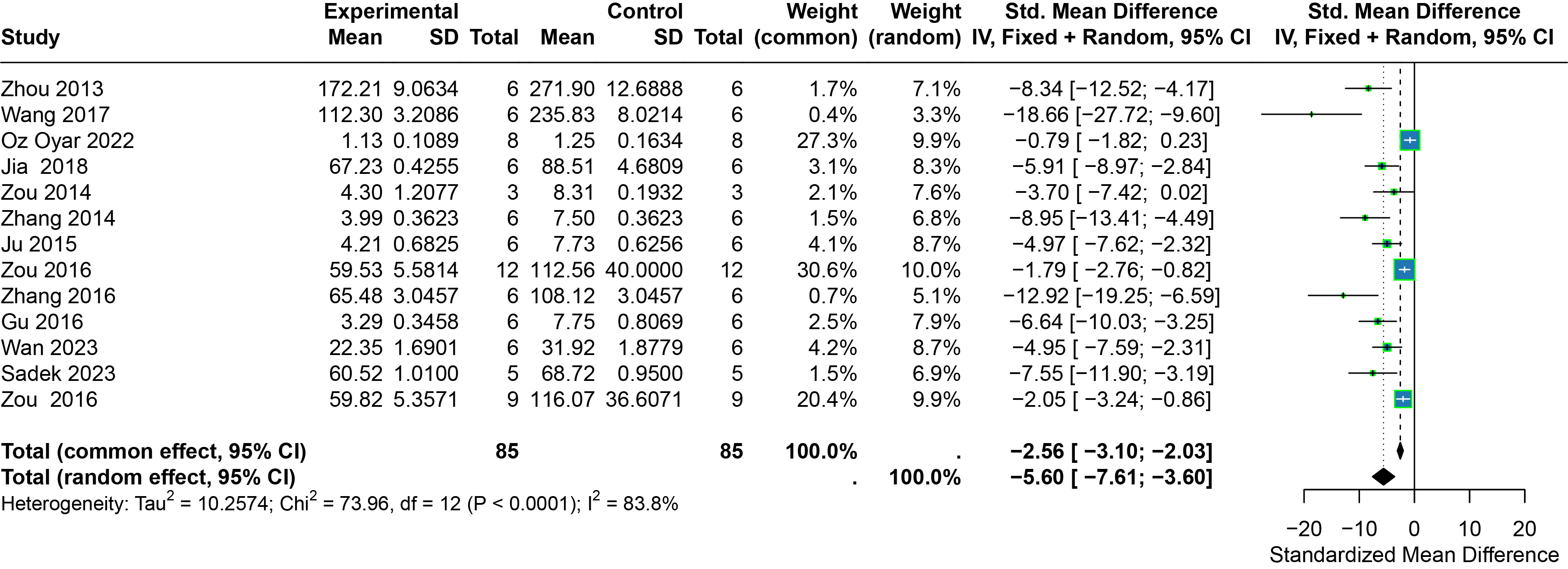


Supplementary Figure 1. Conventional meta-analysis of serum creatinine.


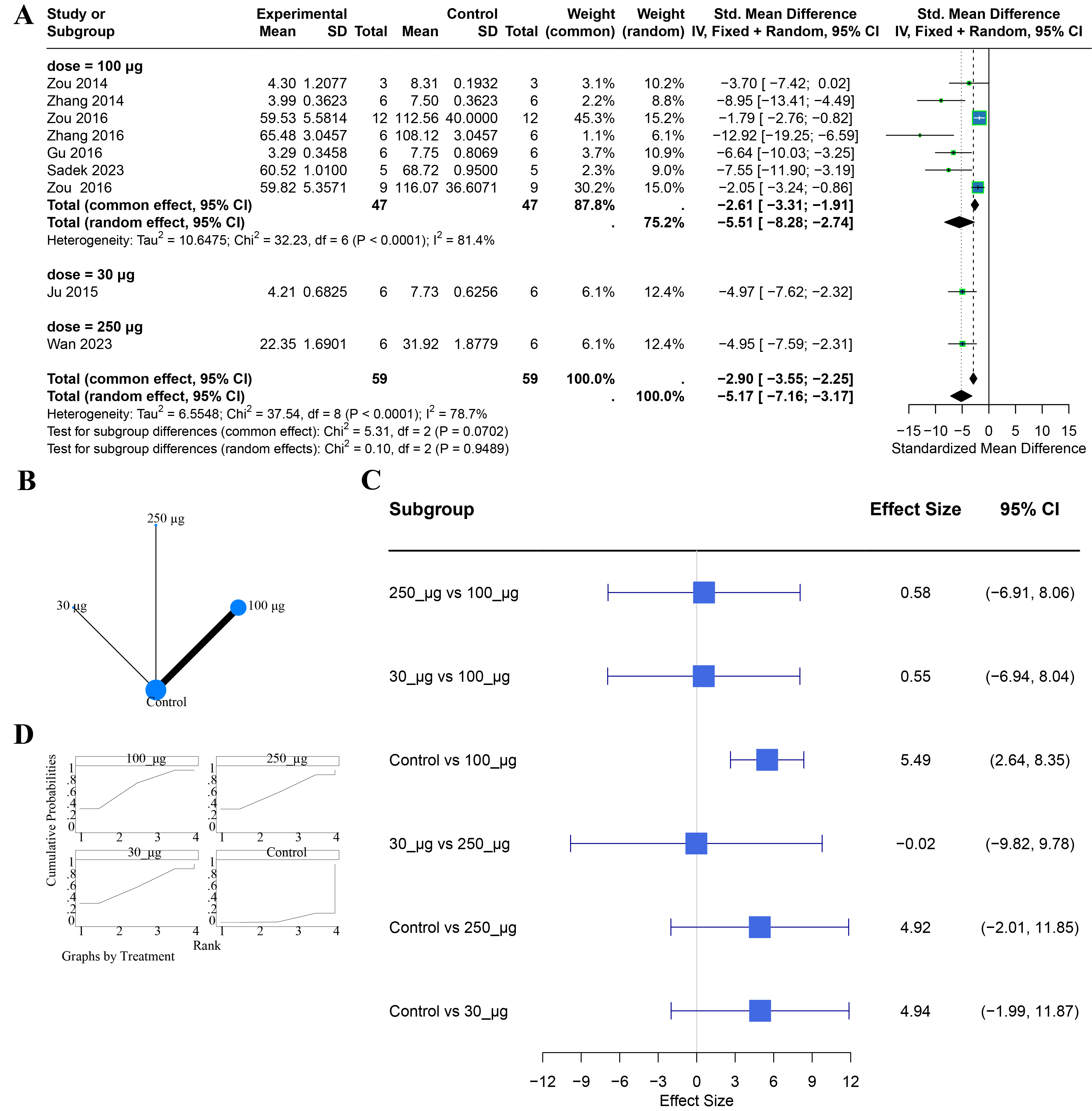


Supplementary Figure 2. Comparative effects of different UCMSC-Exo doses administered via the tail vein on serum creatinine: (A) subgroup analysis; (B) evidence network; (C) SUCRA ranking; (D) network meta-analysis forest plot.


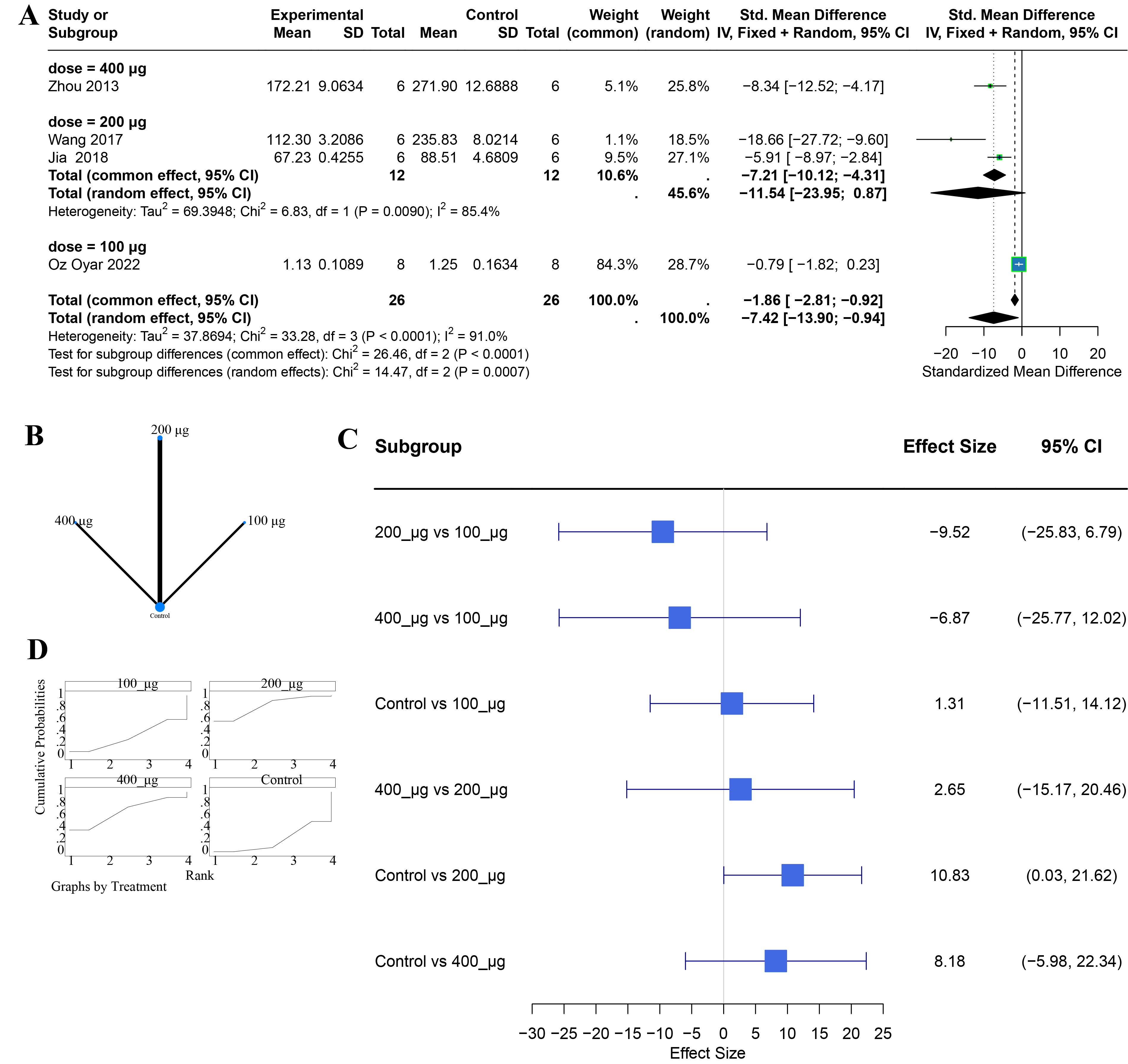


Supplementary Figure 3. Comparative effects of different UCMSC-Exo doses administered via the renal subcapsular route on serum creatinine: (A) subgroup analysis; (B) evidence network; (C) SUCRA ranking; (D) network meta-analysis forest plot.


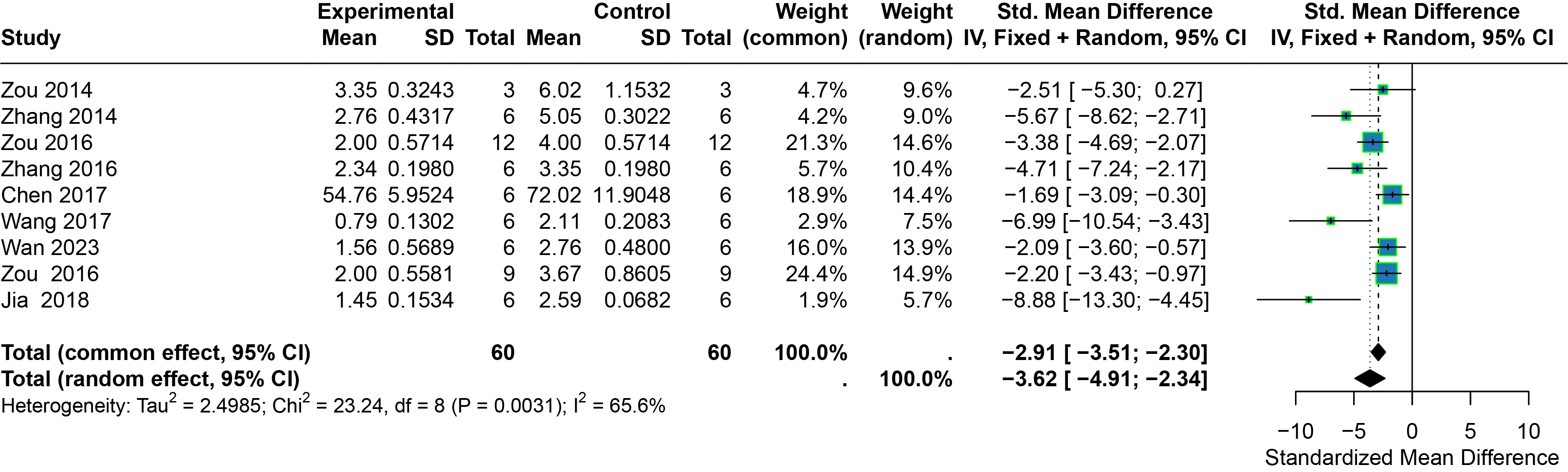


Supplementary Figure 4. Conventional meta-analysis of renal injury scores.


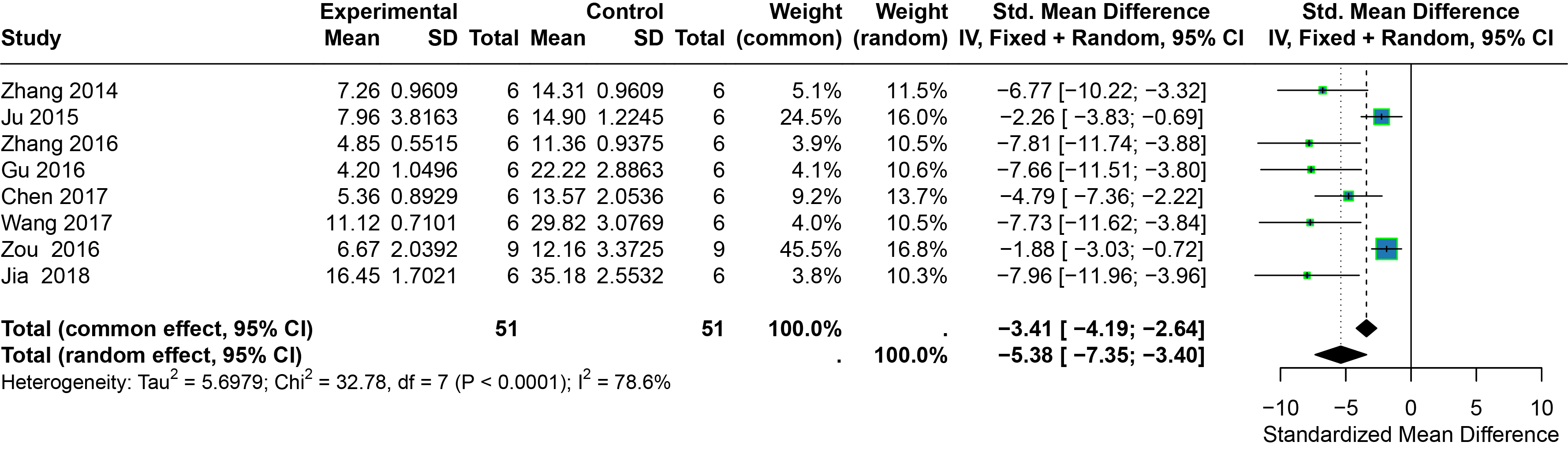


Supplementary Figure 5. Conventional meta-analysis of TUNEL-positive cells.


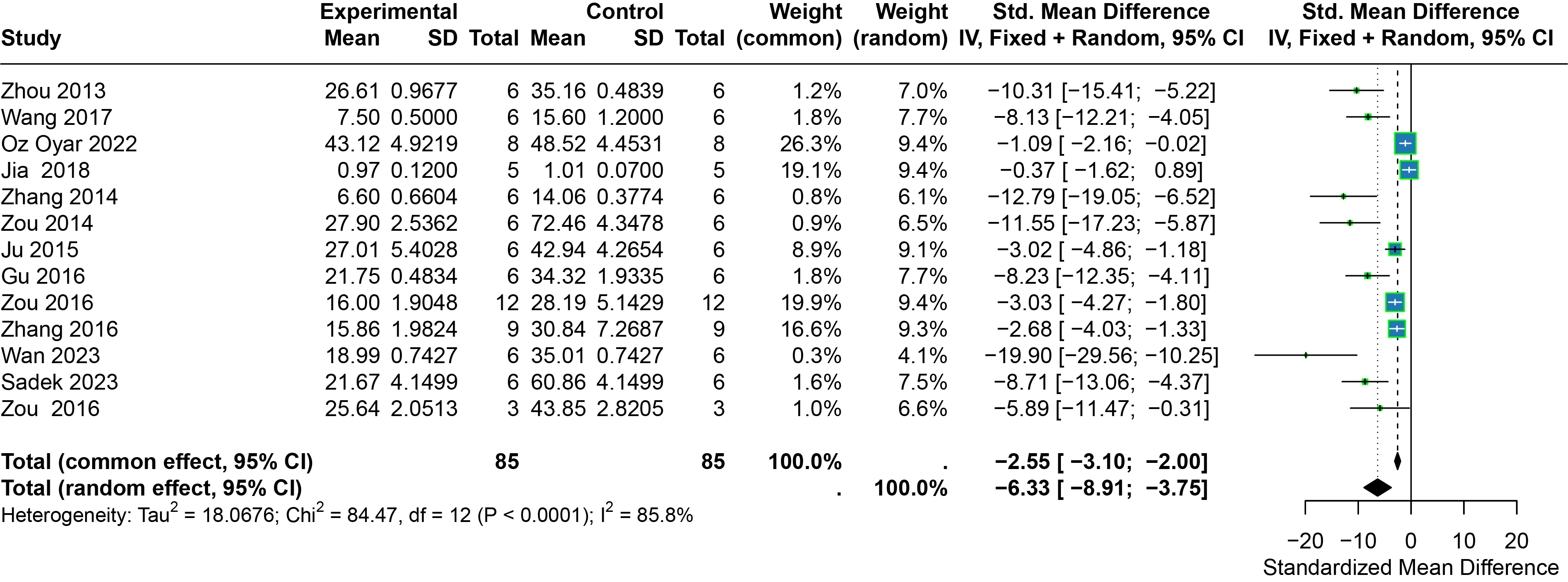


Supplemental Figure 6: Traditional meta-analysis of blood urea nitrogen


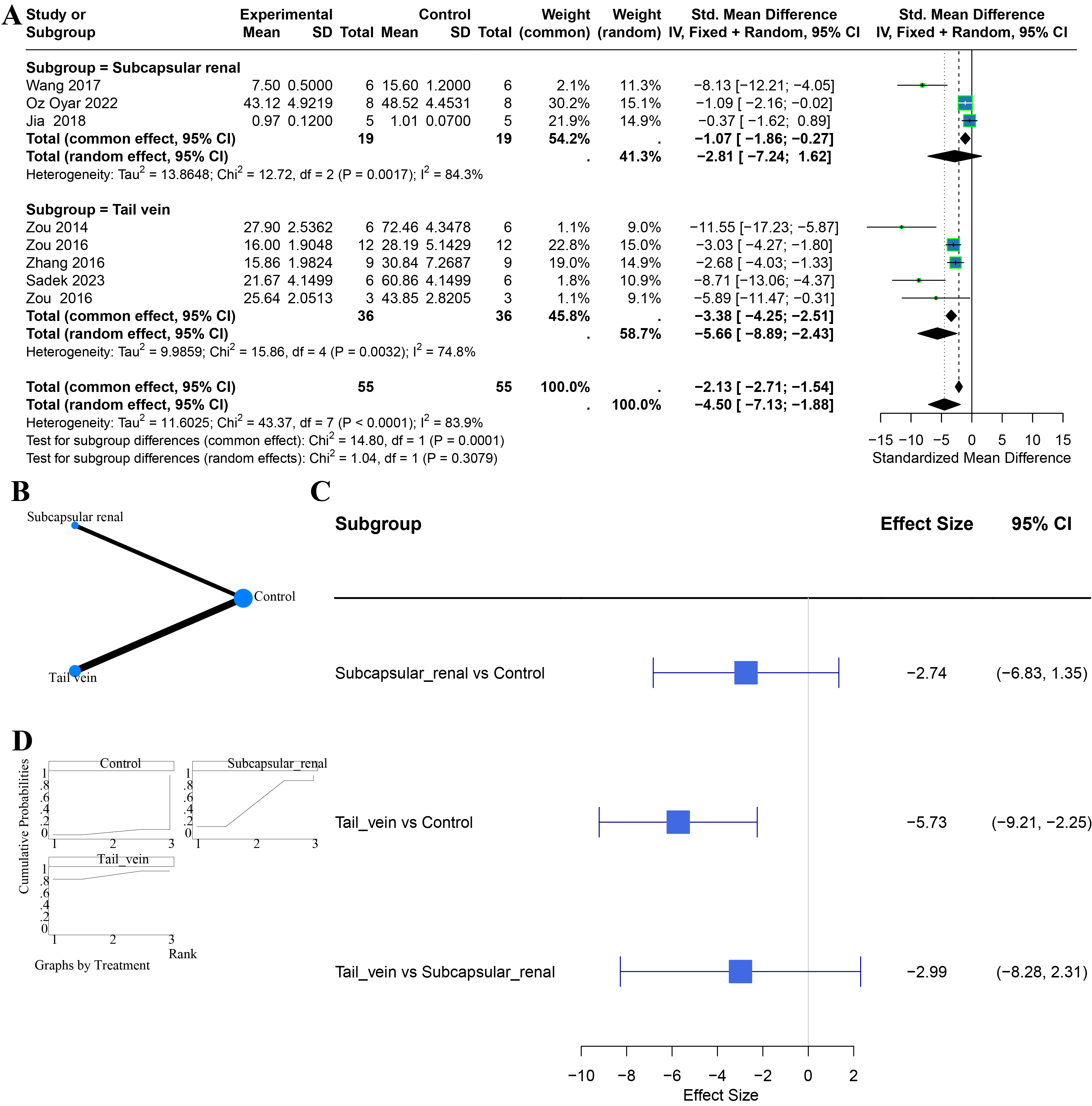


Supplemental Figure 7: Meta-analysis comparing different administration routes at a fixed dose of 100 μg


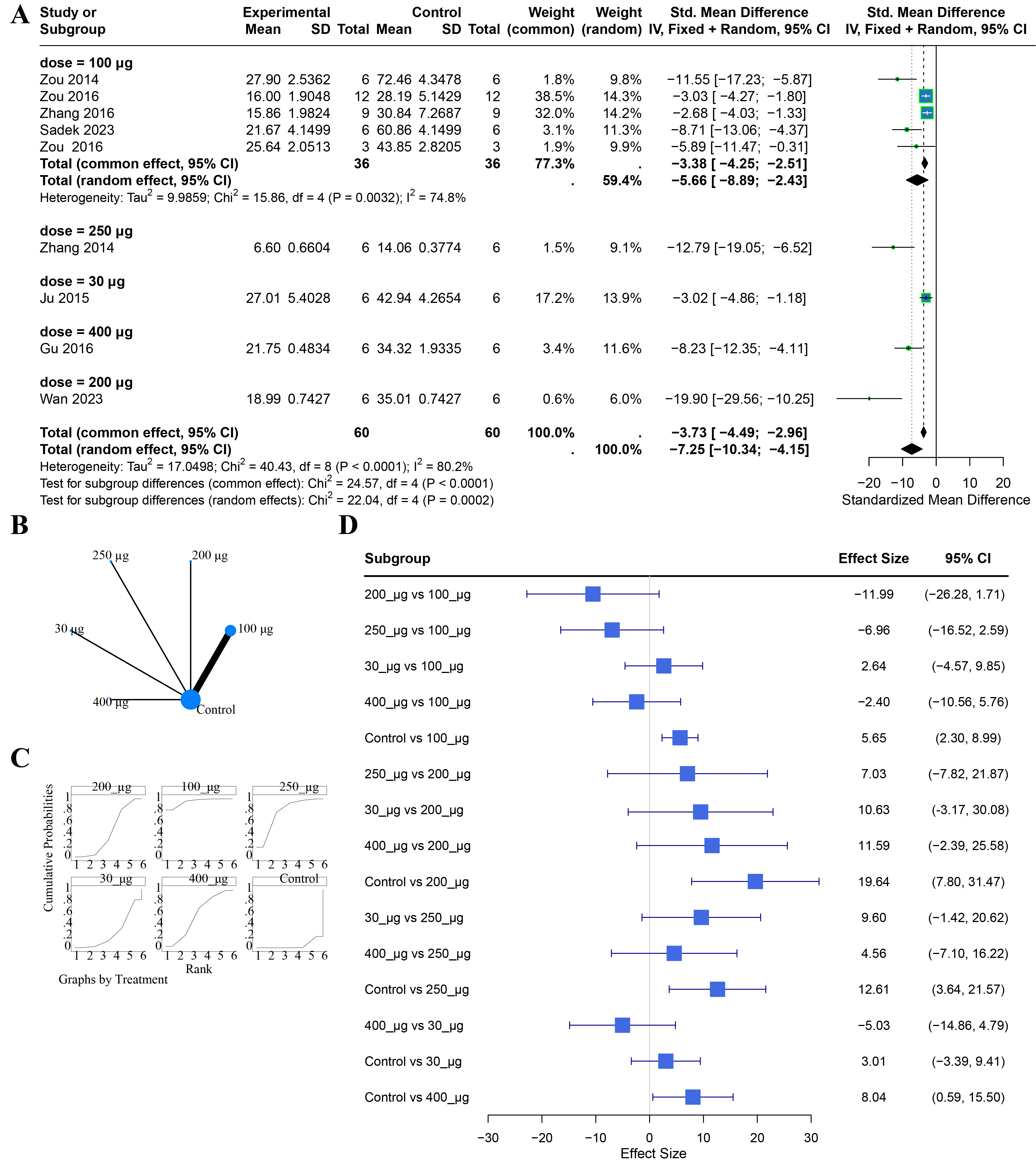


Supplemental Figure 8: Comparative efficacy of different doses of UCMSC-Exo via tail vein injection on blood urea nitrogen levels


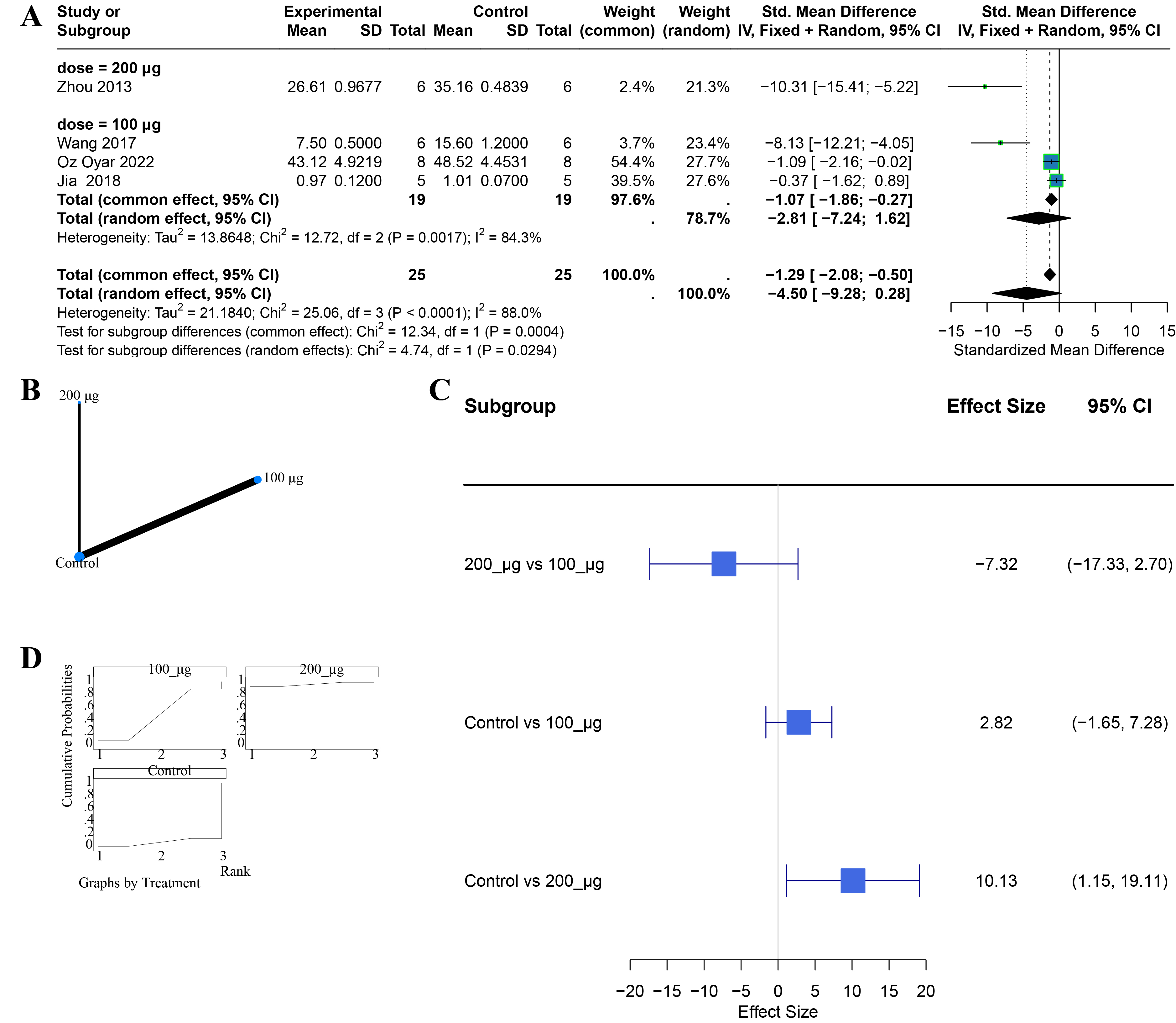


Supplemental Figure 9: Comparative efficacy of different doses of UCMSC-Exo via renal subcapsular injection on blood urea nitrogen levels


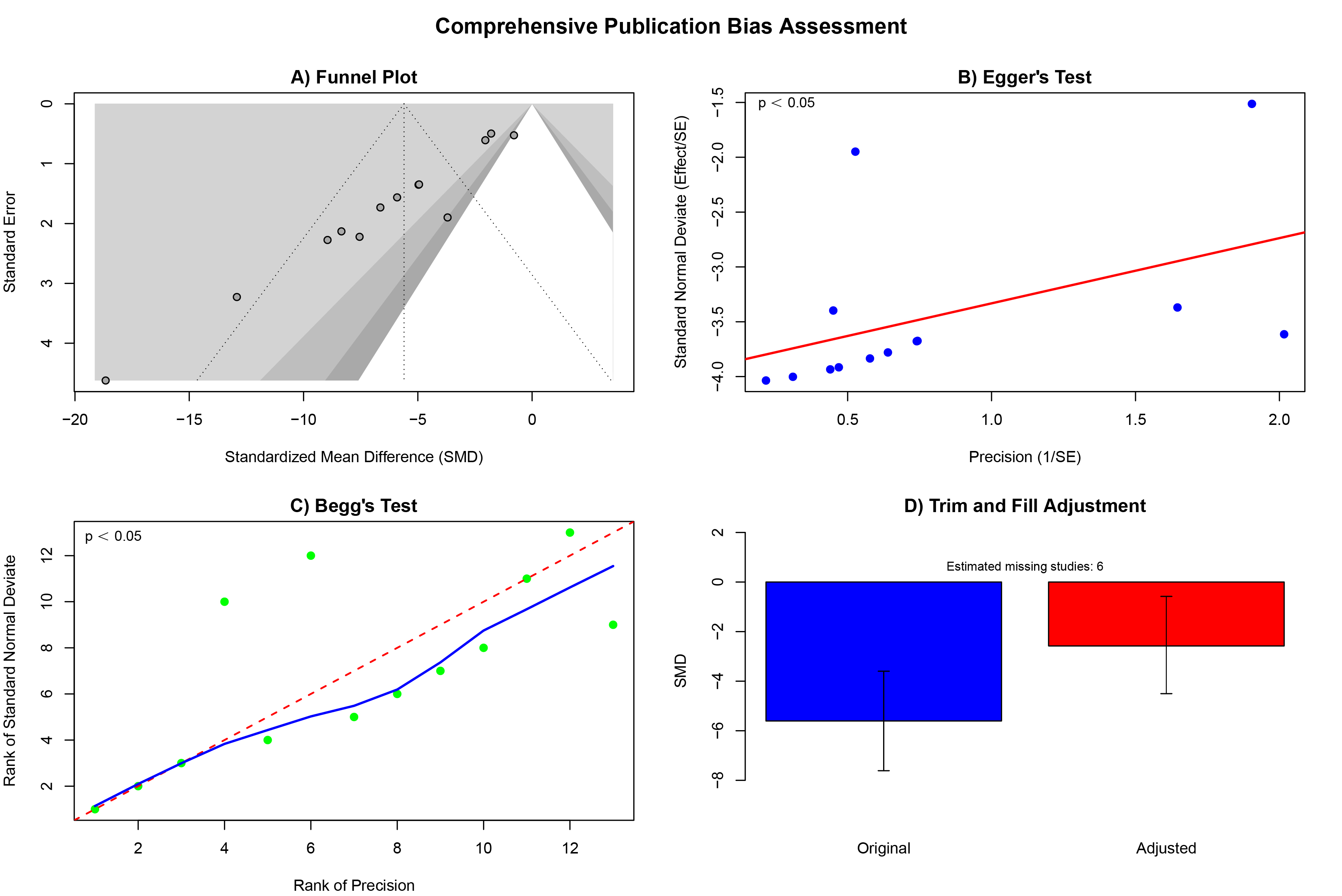


Supplementary Figure 10. Assessment of publication bias based on Scr.


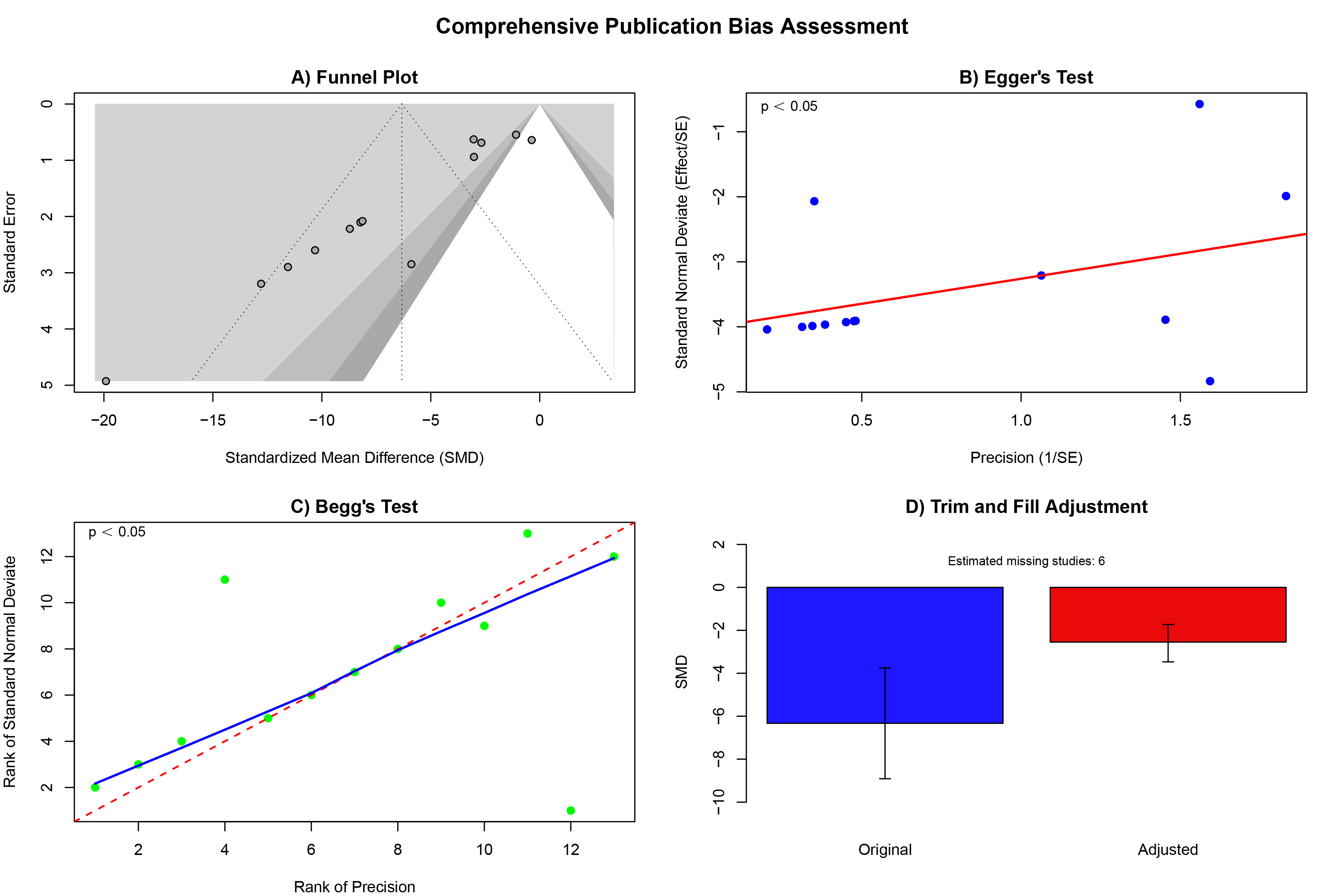


Supplementary Figure 11. Assessment of publication bias based on BUN.
